# Gram Years: A Method to Standardize and Quantify Lifetime Cannabis Consumption

**DOI:** 10.1089/can.2016.0025

**Published:** 2016-09-01

**Authors:** Reagan R. Wetherill, Nathan Hager, Emily Guthier, Teresa R. Franklin

**Affiliations:** Department of Psychiatry, University of Pennsylvania, Philadelphia, Pennsylvania.

**Dear Editor:**

Over the past decade, there has been a dramatic increase in the number of studies and publications examining the effects of cannabis use on the brain and behavior. Despite this rapid increase in research, the field lacks consistency across methodologies and findings.^1^ Indeed, a recent review by Lorenzetti et al. (2014) highlights the methodological differences in the quantification of cannabis exposure and emphasizes the need for a coordinated approach to quantify cannabis use and exposure in human studies. Consequently, we developed a lifetime measurement of cannabis exposure that is based on the well-established “pack years” measure of lifetime cigarette exposure used in nicotine/tobacco research.

The pack years measure from the nicotine/tobacco literature is calculated by dividing the self-reported number of cigarettes smoked per day by 20 cigarettes (i.e., the number of cigarettes in one pack) and then multiplying that number by the number of years the person has smoked cigarettes.^2^ In other words, one pack year is 20 cigarettes smoked/day for 1 year. Pack years smoked is an important clinical care measure, as research indicates that the number of pack years smoked is strongly correlated with risk, severity, and mortality of cigarette smoking-related diseases.^3^ Furthermore, the pack years measure provides a standardized quantification method for researchers to use in their studies that allows for reliable comparisons across studies. Thus, we used the nicotine field's pack years measure to design the gram years measure of lifetime cannabis exposure.

Marijuana is usually purchased in specified amounts. In most cases, users purchase marijuana in grams. Although prices and potency of the cannabis strains vary, a gram of cannabis is a standard amount that users often report using and is the amount typically offered for purchase at medicinal and recreational marijuana dispensaries and shops. As such, we calculate gram years by taking the self-reported number of grams smoked per day and multiply by number of years an individual has been smoking marijuana regularly (grams per day×years of regular cannabis use). Naturally, use reported in ounces must first be converted to grams (e.g., 1/8 ounce, a typical amount of cannabis purchased, is equivalent to 3.5 g). This final number provides a lifetime measure of cannabis exposure that takes into account the quantity of cannabis consumed rather than just the number of days of cannabis use in one's lifetime.

We recognize that there are several limitations to the gram year measure; it does not account for the route of administration (joint, bowl, blunt, bong, etc.), the potency or cannabis strain, or the exact quantified levels from various toxicology tests. Furthermore, it is based on self-reported cannabis use. Despite the limitations, the formula is useful because it provides a lifetime quantification of cannabis exposure that takes into account the quantity of cannabis consumed and it can be modified to account for periods of abstinence. Most importantly, the gram years measure provides a standardized quantification method that allows for reliable comparisons across studies, which are clearly needed in the cannabis literature.
